# Epidemiological Trends of Head and Neck Cancer: A Population-Based Study

**DOI:** 10.1155/2021/1738932

**Published:** 2021-07-14

**Authors:** Kangwen Guo, Weiliang Xiao, Xinggui Chen, Zhenying Zhao, Yuanxiong Lin, Ge Chen

**Affiliations:** ^1^Department of Radiotherapy, Central Hospital of Guangdong Nongken, Zhanjiang, China; ^2^Cancer Center, Affiliated Hospital of Guangdong Medical University, Zhanjiang, China; ^3^Department of Pharmacy, Tianjin Union Medical Center, Tianjin, China; ^4^Department of Intervention, The First Affiliated Hospital of Guangzhou University of Chinese Medicine, Guangzhou, China

## Abstract

**Background:**

Over the past decades, lots of advance have occurred in the prevention, diagnosis, and treatment of head and neck cancer (HNC). However, the contemporaneous incidence and survival trends, on the basis of population-based registry, have not been reported.

**Methods:**

The HNC cancer cases were accessed from the Surveillance, Epidemiology, and End Results (SEER) database. The incidence trend was analyzed by joinpoint analysis, with the survival trend being analyzed by period analysis of relative survival rate (RSR) and Kaplan-Meier analyses. Cox regression analysis was performed to identify the prognostic factors for overall survival.

**Results:**

The general incidence trend of HNC increases slightly, with an average annual percentage change of 0.6%, along with five fluctuating segments. The improvement of net survival over the past decades was showed by increasing 60-month RSR, from 54.1% to 56.0% to 60.9% to 66.8%, which was further confirmed by Kaplan-Meier analyses. Moreover, disparities in incidence and survival patterns can be observed in different subgroups.

**Conclusion:**

A fluctuating incidence pattern and an ever-improving survival were observed in HNC over time.

## 1. Introduction

Head and neck cancer (HNC) covers a wide spectrum of heterogeneous diseases that originate in the head and neck region, including cancers originating from the oral cavity, nasopharynx, oropharynx, larynx, and hypopharynx [1]. Each subtype within this group is associated with unique etiology, epidemiological trends, and therapy [2]. As a major histological type of HNC, head and neck squamous cell carcinoma (HNSCC) is the sixth most common cancer worldwide [3, 4]. Totally, 53,000 new cases and 10,860 HNC-related death are observed in the U.S. in 2019 [5]. According to the estimation of the World Health Organization, 439,000 mouth and oropharynx cancer will be observed in 2030 [6]. The risk factors for HNC include exposure to smoking and alcohol, EB virus infection (nasopharyngeal carcinoma), and HPV infection (especially oropharyngeal cancer) [2]. The epidemiological trend of HNC has shifted significantly due to the increasing incidence of HPV-associated oropharynx cancer [1, 7]. In terms of stage at diagnosis, 29% of cases are categorized as localized cases, with 47% as regional cases and 20% as distant cases [5]. So, it will be of great interest and importance to report the dynamic incidence trend of HNC, which may reflect the impact of changing etiology and the impact of HPV on HNC.

In addition, great advances have been made in the treatment of HNC over the past decades. Currently available treatment methods for HNC include surgery, radiotherapy, chemotherapy, targeted therapy, and immunotherapy, which is generally administrated in combination [8]. Surgeries, both open and microinvasive ones, are the standard treatment for early HNC originating from the oral cavity and early larynx cancer, whereas the intensity-modulated radiation therapy or concurrent chemoradiation is recommended for other early HNC, compared with previous more invasive surgeries and less precise radiotherapy. A majority of HNC patients were diagnosed at an advanced stage (stage III and stage IV), to whom multimodality treatment is explored in recent decades. Currently, chemoradiotherapy represents the standard regimen for these patients, and in patients with bulky disease in which organ preservation is possible, induction chemotherapy followed by cetuximab-radiotherapy is an alternative, compared with previous mono-drug or combinatory chemotherapy [9, 10]. Combinatory use of chemotherapy with a monoclonal antibody targeting epidermal growth factor receptor or immune checkpoint inhibitor represents the standard treatment for metastatic disease [11]. Moreover, immunotherapy is revolutionizing the management of advanced HNC: immunotherapy by anti-PD-1 or anti-PD-L1 antibodies significantly prolongs disease-free survival and overall survival in the second-line setting [12–14]. However, great disparities exist in the treatment response [15]. Although active treatment is given, the disease control rate for advanced HNC is approximately 40% at 5 years, with acute and long-term toxicities as a challenge [2]. Given so much therapeutic development being made over the past decades, the corresponding survival trend of HNC, on the basis of a large sample, remained unreported.

In the era of precision medicine, it is equally important to study the molecular interaction that drives the carcinogenesis of HNC and to study the epidemiological trend of HNC, based on a larger sample size, which may provide clues for bench studies and clinical management. Moreover, previous relevant studies merely based on a small sample, rather than a population-based data resource, limiting their representativeness and generalizability [16–18]. This study is aimed at demonstrating the incidence and survival trend of HNC over the past four decades by analyzing data from the Surveillance, Epidemiology, and End Results (SEER) database.

## 2. Materials and Methods

### 2.1. Case Inclusion

All cases in the current study were accessed from the SEER cancer registry, which was launched by the National Cancer Institute since 1973, with the original nine registries: Connecticut, Detroit, Atlanta, San Francisco-Oakland, Hawaii, Iowa, New Mexico, Seattle-Puget Sound, and Utah. Afterward, the registries were expanded into 13 registries and 18 registries subsequently. The SEER database records the clinicopathological and demographic information, including sex, age, year of diagnosis, tumor stage, survival time, and survival status [19]. Currently, the 18 SEER registries cover approximately 28% of the total population of the USA, representing the most representative data resource for cancer epidemiological studies [20]. Data collection was performed via the SEER∗Stat version 8.3.2 [21]. HNC was defined by the originated site: oral cavity, pharynx, nose, nasal cavity, middle ear, and larynx. Cases diagnosed solely by either autopsy or death certificate are not eligible for the current study. To ensure the quality of the current study, we enrolled cases meeting the following criteria: cases diagnosed by positive histology, cases with active and complete follow-up, and cases with HNC as the first malignancy. Age is categorized into four groups, 0-44, 45-59, 60-74, and 75+. Socioeconomic status (SES) of the county where the patients reside is used as a surrogate for personal SES, after categorizing into three levels based on the same thresholds used in the National Cancer Institute monograph: 10% (low-poverty areas), 10%–19.99% (medium-poverty areas), and >20% (high-poverty areas) [22].

### 2.2. Statistical Analyses

Age-standardized incidence was calculated, and the 2000 US standard population was designated as the reference group. Joinpoint regression analyses were performed to demonstrate the incidence trend over time by the Joinpoint Regression Program version 4.5.0.1. The segmented trend will be estimated by annual percent change (APC) and the incidence trend over the entire study period will be estimated by average annual percent change (AAPC), with corresponding 95% confidence interval (95% CI).

We also calculated the relative survival rates (RSRs), which has been widely adopted to illustrate net survival directly attributed to indexed cancer [13, 23–29]. RSR was calculated on the basis of Ederer II methodology and following expected survival table: U.S. 1970-2015 by individual year (White, Black, Other (AI/API), ages 0-99, all races for Other Unspec 1991+ and Unknown). Specifically, RSRs were calculated by dividing the observed survival (percentage of alive HNC patients at a time point of interest) by the expected survival (estimated percentage of the alive person from the generally comparable population at the same period). The all-cause survival difference was evaluated by the Kaplan-Meier method with the log-rank test for comparison. All these statistical analyses were performed on SEER∗Stat version 8.3.2 and GraphPad Prism 9.0.0 for Windows, GraphPad Software, San Diego, California, USA, https://www.graphpad.com [21]. A two-tailed *P* value less than 0.05 was defined as significant.

## 3. Results

### 3.1. The Incidence Trend of Head and Neck Cancer over the Past Decades

**Figure 1:**
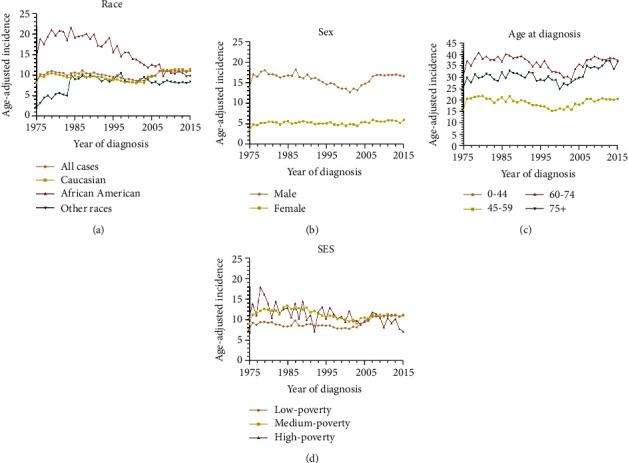
Age-adjusted incidence over the past four decades in head and neck cancer patients in SEER nine registries (a), by race (a), by sex (b), by age at diagnosis (c), and by socioeconomic status (d). SES: socioeconomic status.

Incidence disparities of head and neck cancer (HNC) were observed among different races, sexes, age groups, and SES groups, with a higher incidence in African American, male, senior citizens, and patients from inferior SES (Figure 1). The dynamic and quantitative incidence trend of HNC over the past decades was demonstrated by the joinpoint regression analysis. Between 1975 and 2016, a fluctuating trend was observed, showing an increasing-stagnating-decreasing-increasing-stagnating pattern. Specifically, the incidence of HNC increased before 1977 (APC = 11.04%), remained stable between 1977 and 1990, decreased between 1990 and 2002 (APC = −1.67%), increased 2002 and 2007 (APC = 4.84%), and remained stable thereafter. The average change of incidence over the entire period was 0.6% (Table 1). In terms of race, Caucasians show a fluctuating trend, compared with the increasing-decreasing trend in African American, whose incidence is higher than that of Caucasians. In others, after a sharply increasing trend before 1985, the incidence shows a gradually decreasing trend (APC = −0.48%). With regard to sex, male shows a similarly fluctuating incidence pattern, with a greater amplitude than females, whose incidence is much lower than that of males, with two joinpoints in 1978 and 2000, respectively. In terms of age, there is no significant change in the incidence of HNC in patients aged between 0 and 44 years, compared with other age groups where a fluctuating incidence pattern was observed. In terms of SES, the incidence for patients resided in low-poverty regions and medium-poverty regions show a similarly fluctuating incidence pattern, compared with patients in high-poverty regions, whose incidence shows a consistently decreasing trend. In terms of the stage at diagnosis, after a sharply increasing trend before 1977, the incidence of localized cases decreased afterward in a segmented manner. A fluctuating trend can be seen in both regional and distant cases; of note, the incidence of these two types of cases increases in recent calendar years.

### 3.2. The Survival Changes in Head and Neck Cancer over Time

**Figure 2:**
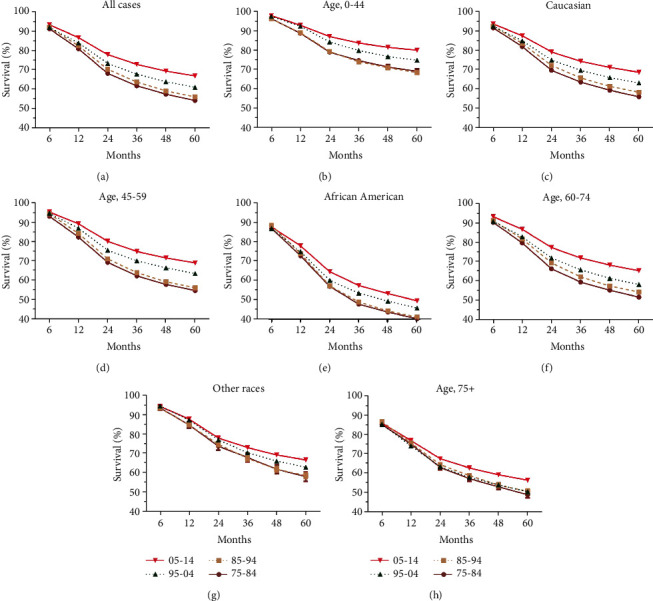
Trends in relative survival rates over past decades for head and neck cancer patients in SEER nine registries (a), by races (c, e, and g) and by age groups (b, d, f, and h).

**Figure 3:**
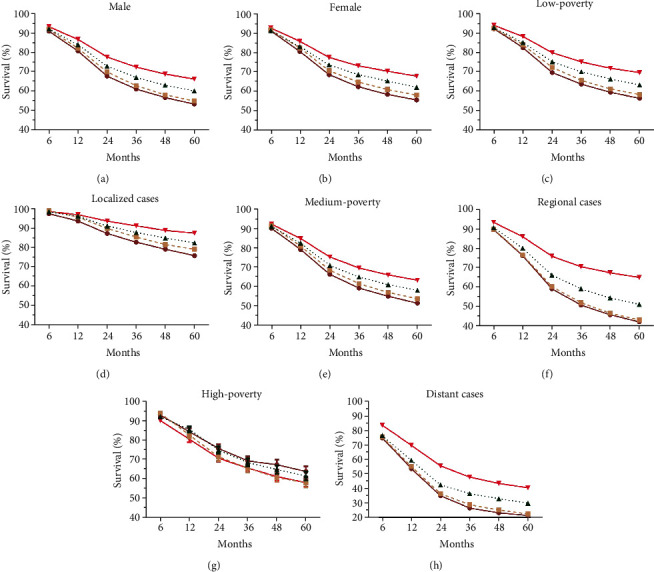
Trends in relative survival rates over past decades for head and neck cancer patients in SEER nine registries by sexes (a, b), by socioeconomic status (c, e, and g), or by stages (d, f, and h).

**Figure 4:**
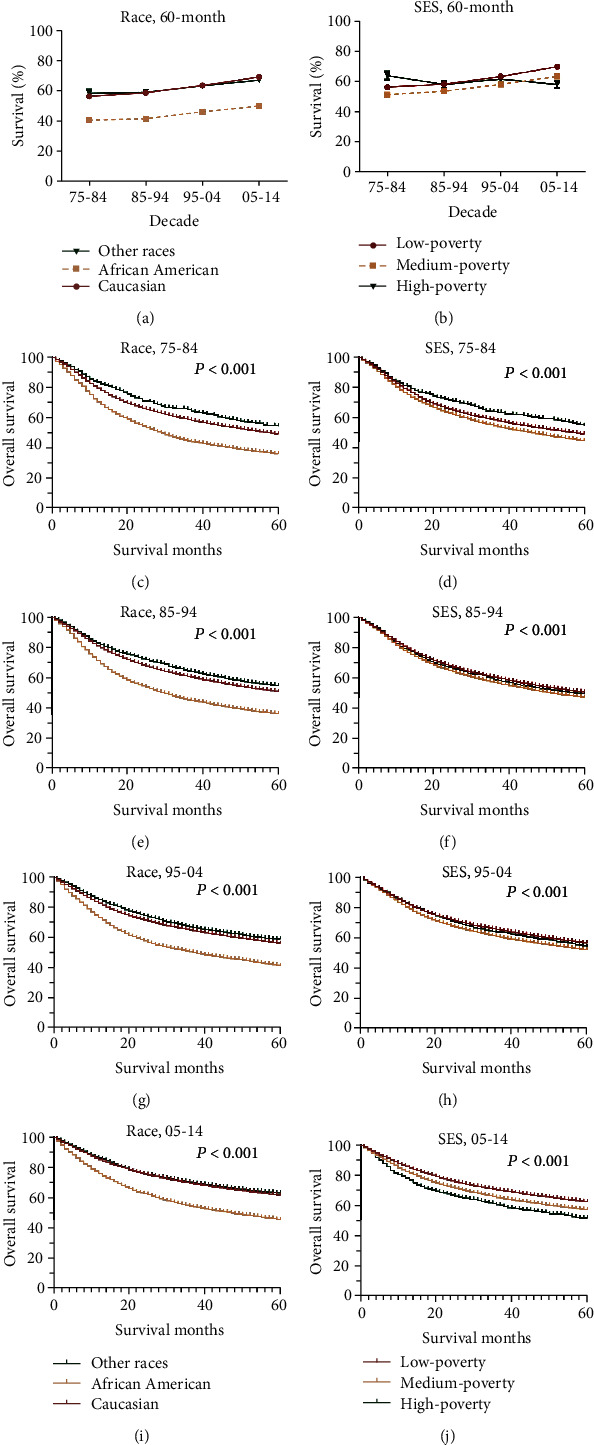
The 60-month relative survival rates by race (a) and SES (b) and Kaplan-Meier survival analyses according to race (c, e, g, and i) and SES (d, f, h, and j) for head and neck cancer patients in each decade between 1975 and 2014.

Significant survival improvement can be observed in all HNC cases and different subgroups (*P* < 0.0001) (Figure 2 and Table 2). Greater improvement was observed in long-term survival, with 5-year survival increasing from 54.1% to 56.0% to 60.9% to 66.8%, compared with the 6-month survival increasing from 91.0% to 91.8% to 91.8% to 93.1% (*P* < 0.0001 for both) (Figure 2(a) and Table 2). And the survival improvement was mainly achieved in the recent two decades (Figure 2(a) and Table 2). After a comparison of survival trends over time by age, all age groups show survival increment, and age-dependent survival disparities were observed, with lower survival in older patients (Figure 2, Table 2). Of note, patients older than 75 years show the lowest RSR as well as a subtle increment, with 6-month RSRs increasing from 84.8% to 86.3% to 84.8% 85.4% and 5-year RSRs increasing from 48.8% to 50.5% to 50.4% to 56.2% (Figure 2(h), Table 2). However, disparities in survival increment can be observed in some subgroups, with modest increment in other races, patients aged older than 75 years, patients resided in high-poverty regions, and localized cases (Figures 2 and 3). In terms of stage, survival improvement can be seen in three different stages in both short-term and long-term survival, with greater improvement in long-term survival and distant cases (Figures 3(d), 3(f), and 3(h) and Table 2). The trends and disparities in net survival showed by RSR can also be confirmed in overall survival by Kaplan-Meier analyses (Supplementary Figure 1, 2). In terms of sex, significant survival improvement can be seen in both sexes; however, female shows generally better survival than males, in both short-term and long-term survival (Table 2). In terms of race, significant survival improvement was observed in all races, with generally better survival in Caucasians and others (Figure 4(a)). More importantly, survival increment is much subtler in African Americans. Similarly, with regard to socioeconomic status (SES), significant survival improvement can be observed in different SES groups, with greater increment in low-poverty and medium-poverty groups (Supplementary Figure 1C, E). Moreover, better survival was observed in low-poverty and medium-poverty groups (Figure 4(b)). Being different from other cancer types where the survival disparities among races and patients from different SES kept widening simultaneously, the survival disparities among races in HNC kept narrowing comparing with the widening survival gaps among different SES groups since the second decade (Figures 4(c), 4(e), 4(g), and 4(i)) [23–28, 30–33].

## 4. Discussion

The current study demonstrated the general incidence and survival trends of HNC over the past decades on the basis of a population-based SEER database. To the best of our knowledge, this is the first study that provides representative and generalizable data, which may aid in estimating the disease burden for the pharmaceutic industry and healthcare system and ultimately improve patients' survival.

Here, we demonstrated a fluctuating incidence trend and a generally increasing survival trend of HNC. Previous studies have identified risk factors for HNC, including excessive alcohol intake, smoking, EBV infection, HPV infection, areca nut, and airborne pollutants [34, 35]. However, the detailed mechanism for HNC carcinogenesis, including the signaling pathway that subsequently drives normal tissue to the precancerous lesion to the cancerous lesion, remained largely unknown. Interestingly, the etiological spectrum kept evolving. Previously, 75% of HNC is believed to be associated with exposure to smoking and alcohol compared with the remaining 25% HPV-related cases [7, 36]. In recent years, due to the smoking cessation program and the increasing incidence of HPV infection in the USA, a decrease in the incidence of HPV negative HNC was observed, whereas the incidence of HPV-positive HNC kept increasing [37]. The pathogenic role of HPV infection is more predominant in oropharyngeal carcinoma. The prevalence of HPV+ oropharyngeal increased sharply over time, with from 40.5% before 2000, to 64.3% between 2000 and 2004, and 72.2% between 2005 and 2009 (*P* < 0.001) [38]. A population-based Danish study shows that increase of oropharyngeal squamous cell carcinoma is mainly attributed to the increased prevalence of HPV+ cases. Given the pivotal role of HPV in oropharyngeal carcinoma, a revision of TNM staging system on HPV+ cases was proposed and validated to be prognostic, which is incorporated in the 8^th^ staging system [39, 40]. The decreasing trend between 1990 and 2002 may be caused by the decreasing alcohol intake since the 1980s, whereas the increasing trend since the 2000s may be attributed to the higher alcohol intake [41, 42]. The generally lower incidence and better prognosis of HNC in females than males may imply the protective role of estrogen, and the exogenous estrogen may lower the risk of developing HNC [6, 43]. The different incidence patterns between patients younger than 44 years and patients older than 44 years implied increased accumulative risk factor exposure and thus more genetic mutation accumulated and increased DNA methylation at age-related sites in the older generation [44, 45]. The ever-increasing general incidence trend of HNC patients from inferior SES regions may be attributed to their increased exposure to risk factors: alcohol, smoking, and HPV infection [46–48]. The increased incidence of advanced-stage cases here may imply the more sensitive imaging detection adopted, instead of a more dismal clinical scenario [49].

The relative survival of HNC kept increasing over the past decades, especially in long-term survival, with survival disparities among age groups, sexes, races, SES groups, and stages. Additionally, similar trends can also be seen in all-cause survival by Kaplan-Meier analyses. In terms of sex, the prognosis in females is generally better than that of males, implying an inhibitory role of estrogen in the growth of HNC [43]. Of note, the ever-widening survival gap among patients from different SES groups implies the greater impact of social status on determining the treatment availability and ultimately the patients' prognosis [13, 23–28, 30–33, 50]. With regard to the stage, survival improvement can be seen in all three stages, with greater improvement in advanced cases (regional and distant cases). For localized cases, the standardized treatment for HNC is generally multimodal, with surgery followed by chemoradiotherapy for oral cavity cancer and primary chemoradiotherapy for pharynx and larynx cancer [34]. The survival improvement observed here was accomplished by the revolutionizing therapeutic landscape for HNC over the past decades. Specifically, the advent of intensity-modulated radiotherapy and proton radiotherapy promotes the more precise radiation delivery, better sparing of adjacent normal tissue, and ultimately superior cancer control and posttreatment quality of life [8, 51, 52]. Moreover, concurrent chemoradiotherapy constitutes an absolute 6.5% increment in five-year survival compared with radiotherapy alone, which may be attributed to the radio-sensitizing effect of platinum-based chemotherapy [53]. The addition of Cetuximab, an anti-EGFR monoclonal antibody, may help improve patients' prognosis in different settings, including first-line chemotherapy in metastatic or refractory settings and curative settings [54, 55]. Robotic surgery for oropharyngeal cancer was approved by the FDA in 2009 and shows comparable survival outcomes with radiotherapy [56, 57]. Evolving management algorithm for advanced cases drives their prognosis improvement [34]. Immunotherapy by anti-PD-1 or anti-PD-L1 antibodies was approved in the second-line setting, and the current effort focuses on identifying biomarkers for selecting patients that can benefit more from the immunotherapy [12–14]. For recurrent or distant cases which is amenable to previous local treatment, resection, radiation, or limited-volume irradiation followed by observation is suggested. Cases relapsed from platinum-based therapy are subjected to nivolumab or pembrolizumab. For recurrent or distant cases that are not amenable to local treatment, systematic treatment is indicated: cases without PD-L1 expression are indicated for chemotherapy plus cetuximab; cases with PD-L1 expression and lower tumor burden are indicated for pembrolizumab monotherapy; cases with PD-L1 expression and high tumor burden are indicated for chemotherapy plus pembrolizumab. Participation in clinical trials is also a possibility for these patients.

Despite novel findings, the current study should be interpreted in the context of limitations. First, the conclusions here may be biased by the retrospective nature of the current study. Second, all cases here are from the SEER database, and therefore, the conclusion here may be biased if there is any underregistration or miscoding during data proceeding. Third, due to the fact that all the data here are based on the SEER databases and the etiological factors for HNC vary among regions, all the results here merely represent the landscape of the U.S. Thus, cautions are suggested while applying results and conclusion here to regions other than the U.S.

In conclusion, the current study, on the basis of a population-based database, provides generalizable epidemiological data on the incidence and survival trends of HNC over the past decades. Analyzing the incidence and survival trends and the associated disparities may help predict future trends, design healthcare policy to better balance these disparities, and ultimately improve the clinical management of HNC.

## Figures and Tables

**Table 1:** Joinpoint analyses for patients diagnosed with head and neck cancer between 1975 to 2016 in SEER nine registries.

Characteristics	Year	APC (95% CI)	AAPC (95% CI)
Overall	1975 to 1977	11.0^∗^ (3.1, 19.6)	0.6^∗^ (0.1, 1.1)
	1977 to 1990	-0.1 (-0.5, 0.2)	
	1990 to 2002	-1.7^∗^ (-2.1, -1.2)	
	2002 to 2007	4.8^∗^ (2.5, 7.3)	
	2007 to 2016	0.2 (-0.4, 0.8)	
Race			
Caucasian	1975 to 1978	8.2^∗^ (4, 12.7)	0.8^∗^ (0.1, 1.5)
	1978 to 1984	-1.8 (-3.7, 0.1)	
	1984 to 1987	2 (-5.1, 9.7)	
	1987 to 2002	-1.5^∗^ (-1.8, -1.1)	
	2002 to 2008	5.9^∗^ (4.2, 7.7)	
	2008 to 2016	0 (-0.7, 0.7)	
African American	1975 to 1984	2.5^∗^ (0.5, 4.6)	-1.5^∗^ (-1.9, -1)
	1984 to 2016	-2.5^∗^ (-2.8, -2.3)	
Other	1975 to 1985	12.8^∗^ (8.8, 16.9)	2.6^∗^ (1.7, 3.5)
	1985 to 2016	-0.5^∗^ (-0.8, -0.2)	
Sex			
Male	1975 to 1977	9.9^∗^ (1.2, 19.3)	0.4 (-0.1, 0.9)
	1977 to 1990	-0.4 (-0.8, 0.1)	
	1990 to 2002	-2.0^∗^ (-2.5, -1.6)	
	2002 to 2008	4.6^∗^ (3.1, 6.1)	
	2008 to 2016	-0.2 (-0.7, 0.4)	
Female	1975 to 1978	11.1^∗^ (1.1, 22.1)	1.0^∗^ (0.2, 1.7)
	1978 to 2000	-0.4^∗^ (-0.8, 0)	
	2000 to 2016	1.1∗ (0.6, 1.7)	
Age			
0-44	1975 to 1978	7.8 (-3.2, 20.1)	0.9 (-0.1, 2)
	1978 to 2003	0.2 (-0.1, 0.6)	
	2003 to 2008	4.2 (-1, 9.6)	
	2008 to 2016	-1.3 (-2.9, 0.3)	
45–59	1975 to 1977	16.8^∗^ (1.3, 34.8)	0.5 (-0.3, 1.3)
	1977 to 2001	-1.4^∗^ (-1.7, -1.1)	
	2001 to 2008	3.5^∗^ (1.6, 5.4)	
	2008 to 2016	0 (-1.2, 1.1)	
60-74	1975 to 1978	6.5^∗^ (0.2, 13.2)	0.4 (-0.3, 1.2)
	1978 to 1990	0 (-0.7, 0.7)	
	1990 to 2003	-1.9^∗^ (-2.5, -1.3)	
	2003 to 2007	6.0^∗^ (0.7, 11.5)	
	2007 to 2016	0.1 (-0.6, 0.9)	
75+	1975 to 1989	0.9^∗^ (0.1, 1.7)	0.6 (0, 1.2)
	1989 to 2002	-1.5^∗^ (-2.3, -0.6)	
	2002 to 2008	4.6^∗^ (1.6, 7.7)	
	2008 to 2016	0.3 (-1, 1.7)	
SES			
Low-poverty	1975 to 1977	9 (-3.3, 22.9)	0.9^∗^ (0.2, 1.5)
	1977 to 2002	-0.7^∗^ (-0.9, -0.5)	
	2002 to 2007	5.9^∗^ (3.5, 8.4)	
	2007 to 2016	0.6^∗^ (0.1, 1.2)	
Medium-poverty	1975 to 1977	14.2^∗^ (3.1, 26.6)	0.4 (-0.3, 1.1)
	1977 to 1988	0.6 (-0.2, 1.3)	
	1988 to 2003	-2.0^∗^ (-2.4, -1.6)	
	2003 to 2007	4.4 (-0.1, 9)	
	2007 to 2016	-0.4 (-1.2, 0.3)	
High-poverty	1975 to 2016	-1.1^∗^ (-1.4, -0.7)	-1.1^∗^ (-1.4, -0.7)
Stage			
Localized	1975 to 1977	13.7 (-1.7, 31.4)	-0.7 (-1.9, 0.6)
	1977 to 1990	-0.4 (-1.1, 0.3)	
	1990 to 2002	-2.0^∗^ (-2.7, -1.4)	
	2002 to 2005	-8.1 (-19.9, 5.5)	
	2005 to 2015	0.2 (-0.9, 1.4)	
Regional	1975 to 1987	2.2^∗^ (1.2, 3.2)	0.4 (-0.7, 1.5)
	1987 to 2002	-0.5 (-1.1, 0.1)	
	2002 to 2005	-5.5 (-18.1, 9)	
	2005 to 2015	1.4^∗^ (0.3, 2.4)	
Distant	1975 to 1979	5.3 (-1.9, 12.9)	1.0^∗^ (0.2, 1.9)
	1979 to 1998	-2.4^∗^ (-3.2, -1.7)	
	1998 to 2015	4.0^∗^ (3.2, 4.8)	

APC: annual percent change; AAPC: average annual percent change; CI: confidence interval; SES: socioeconomic status. ^∗^ indicates either APC or AAPC is significantly different from zero (*P* < 0.05).

**Table 2:** Summary for 6-, 12-, 24-, 36-, 48-, and 60-month RSR, SEM, and number of cases in patients diagnosed with head and neck cancer from the SEER nine registry sites in each decade.

Groups	RSR	Calendar period			
		75-84	85-94	95-04	05-14
All	6-month	91.0 ± 0.2 (19301)	91.8 ± 0.2 (21956)^∗∗∗^	91.8 ± 0.2 (22830)	93.1 ± 0.2 (32965)^∗∗∗^
	12-month	80.7 ± 0.3 (19301)	82.3 ± 0.3 (21956)^∗∗∗^	83.8 ± 0.3 (22830)^∗∗∗^	86.5 ± 0.2 (32965)^∗∗∗^
	24-month	68.1 ± 0.4 (19301)	70.2 ± 0.3 (21956)^∗∗∗^	73.2 ± 0.3 (22830)^∗∗∗^	77.7 ± 0.3 (32965)^∗∗∗^
	36-month	61.6 ± 0.4 (19301)	63.6 ± 0.4 (21956)^∗∗∗^	67.7 ± 0.3 (22830)^∗∗∗^	72.7 ± 0.3 (32965)^∗∗∗^
	48-month	57.4 ± 0.4 (19301)	59.0 ± 0.4 (21956)^∗∗∗^	63.8 ± 0.4 (22830)^∗∗∗^	69.3 ± 0.3 (32965)^∗∗∗^
	60-month	54.1 ± 0.4 (19301)	56.0 ± 0.4 (21956)^∗∗∗^	60.9 ± 0.4 (22830)^∗∗∗^	66.8 ± 0.3 (32965)^∗∗∗^

0-44	6-month	96.3 ± 0.5 (1501)	96.2 ± 0.4 (2173)^∗∗∗^	97.4 ± 0.3 (2562)^∗∗∗^	97.8 ± 0.3 (2865)^∗∗∗^
	12-month	88.8 ± 0.8 (1501)	89.0 ± 0.7 (2173)^∗∗∗^	92.4 ± 0.5 (2562)^∗∗∗^	92.9 ± 0.5 (2865)^4^
	24-month	79.1 ± 1.1 (1501)	79.4 ± 0.9 (2173)^∗∗∗^	84.3 ± 0.7 (2562)^∗∗∗^	87.1 ± 0.6 (2865)^∗∗∗^
	36-month	74.7 ± 1.1 (1501)	74.0 ± 1.0 (2173)^∗∗∗^	79.9 ± 0.8 (2562)^∗∗∗^	83.7 ± 0.7 (2865)^∗∗∗^
	48-month	71.4 ± 1.2 (1501)	71.1 ± 1.0 (2173)^∗∗∗^	76.8 ± 0.9 (2562)^∗∗∗^	81.6 ± 0.8 (2865)^∗∗∗^
	60-month	69.3 ± 1.2 (1501)	68.6 ± 1.0 (2173)^∗∗∗^	74.9 ± 0.9 (2562)^∗∗∗^	80.1 ± 0.8 (2865)^∗∗∗^

45-59	6-month	93.0 ± 0.3 (6879)	94.3 ± 0.3 (6667)^∗∗∗^	94.6 ± 0.3 (7931)^∗∗∗^	95.2 ± 0.2 (12238)^∗∗∗^
	12-month	82.4 ± 0.5 (6879)	84.3 ± 0.5 (6667)^∗∗∗^	86.9 ± 0.4 (7931)^∗∗∗^	89.2 ± 0.3 (12238)^∗∗∗^
	24-month	69.5 ± 0.6 (6879)	71.3 ± 0.6 (6667)^∗∗∗^	75.7 ± 0.5 (7931)^∗∗∗^	80.3 ± 0.4 (12238)^∗∗∗^
	36-month	62.6 ± 0.6 (6879)	64.3 ± 0.6 (6667)^∗∗∗^	70.3 ± 0.5 (7931)^∗∗∗^	75.1 ± 0.4 (12238)^∗∗∗^
	48-month	58.2 ± 0.6 (6879)	59.6 ± 0.6 (6667)^∗∗∗^	66.8 ± 0.6 (7931)^∗∗∗^	71.8 ± 0.4 (12238)^∗∗∗^
	60-month	55.1 ± 0.6 (6879)	56.5 ± 0.6 (6667)^∗∗∗^	63.8 ± 0.6 (7931)^∗∗∗^	69.2 ± 0.5 (12238)^∗∗∗^

60-74	6-month	90.3 ± 0.3 (8344)	91.2 ± 0.3 (9573)^∗∗∗^	90.8 ± 0.3 (8279)^∗∗∗^	93.2 ± 0.2 (12448)^∗∗∗^
	12-month	79.7 ± 0.5 (8344)	81.8 ± 0.4 (9573)^∗∗∗^	82.9 ± 0.4 (8279)^∗∗∗^	86.7 ± 0.3 (12448)^∗∗∗^
	24-month	66.5 ± 0.6 (8344)	69.6 ± 0.5 (9573)^∗∗∗^	72.1 ± 0.5 (8279)^∗∗∗^	77.4 ± 0.4 (12448)^∗∗∗^
	36-month	59.7 ± 0.6 (8344)	62.4 ± 0.5 (9573)^∗∗∗^	66.0 ± 0.6 (8279)^∗∗∗^	72.1 ± 0.4 (12448)^∗∗∗^
	48-month	55.5 ± 0.6 (8344)	57.7 ± 0.6 (9573)^∗∗∗^	61.6 ± 0.6 (8279)^∗∗∗^	68.4 ± 0.5 (12448)^∗∗∗^
	60-month	52.0 ± 0.6 (8344)	54.6 ± 0.6 (9573)^∗∗∗^	58.5 ± 0.6 (8279)^∗∗∗^	65.6 ± 0.5 (12448)^∗∗∗^

75+	6-month	84.8 ± 0.8 (2577)	86.3 ± 0.7 (3543)^∗∗∗^	84.8 ± 0.6 (4058)^∗∗∗^	85.4 ± 0.5 (5414)^∗∗∗^
	12-month	74.7 ± 1.0 (2577)	75.3 ± 0.8 (3543)^∗∗∗^	73.8 ± 0.8 (4058)^∗∗∗^	76.5 ± 0.7 (5414)^∗∗∗^
	24-month	62.8 ± 1.2 (2577)	64.2 ± 1.0 (3543)^∗∗∗^	63.1 ± 0.9 (4058)^∗∗∗^	67.3 ± 0.8 (5414)^∗∗∗^
	36-month	57.0 ± 1.3 (2577)	58.6 ± 1.1 (3543)^∗∗∗^	57.7 ± 1.0 (4058)^∗∗∗^	62.5 ± 0.9 (5414)^∗∗∗^
	48-month	52.9 ± 1.4 (2577)	53.8 ± 1.2 (3543)^∗∗∗^	53.9 ± 1.1 (4058)^∗∗∗^	59.0 ± 1.0 (5414)^∗∗∗^
	60-month	48.8 ± 1.5 (2577)	50.5 ± 1.2 (3543)^∗∗∗^	50.4 ± 1.1 (4058)^∗∗∗^	56.2 ± 1.1 (5414)^∗∗∗^

Male	6-month	91.0 ± 0.3 (14100)	91.9 ± 0.2 (15724)^∗∗∗^	92.1 ± 0.2 (16047)^∗∗∗^	93.3 ± 0.2 (23913)^∗∗∗^
	12-month	80.8 ± 0.4 (14100)	82.2 ± 0.3 (15724)^∗∗∗^	84.1 ± 0.3 (16047)^∗∗∗^	86.8 ± 0.2 (23913)^∗∗∗^
	24-month	67.9 ± 0.4 (14100)	70.0 ± 0.4 (15724)^∗∗∗^	73.0 ± 0.4 (16047)^∗∗∗^	77.8 ± 0.3 (23913)^∗∗∗^
	36-month	61.2 ± 0.5 (14100)	63.0 ± 0.4 (15724)^∗∗∗^	67.2 ± 0.4 (16047)^∗∗∗^	72.5 ± 0.3 (23913)^∗∗∗^
	48-month	56.9 ± 0.5 (14100)	58.2 ± 0.4 (15724)^∗∗∗^	63.2 ± 0.4 (16047)^∗∗∗^	68.9 ± 0.3 (23913)^∗∗∗^
	60-month	53.5 ± 0.5 (14100)	55.1 ± 0.5 (15724)^∗∗∗^	60.3 ± 0.4 (16047)^∗∗∗^	66.4 ± 0.4 (23913)^∗∗∗^

Female	6-month	91.1 ± 0.4 (5201)	91.6 ± 0.4 (6232)^∗∗∗^	91.3 ± 0.4 (6783)^∗∗∗^	92.6 ± 0.3 (9052)^∗∗∗^
	12-month	80.6 ± 0.6 (5201)	82.4 ± 0.5 (6232)^∗∗∗^	83.0 ± 0.5 (6783)^∗∗∗^	85.8 ± 0.4 (9052)^∗∗∗^
	24-month	68.8 ± 0.7 (5201)	70.8 ± 0.6 (6232)^∗∗∗^	73.8 ± 0.6 (6783)^∗∗∗^	77.6 ± 0.5 (9052)^∗∗∗^
	36-month	62.6 ± 0.7 (5201)	65.0 ± 0.7 (6232)^∗∗∗^	68.8 ± 0.6 (6783)^∗∗∗^	73.3 ± 0.5 (9052)^∗∗∗^
	48-month	58.7 ± 0.7 (5201)	61.2 ± 0.7 (6232)^∗∗∗^	65.5 ± 0.6 (6783)^∗∗∗^	70.6 ± 0.6 (9052)^∗∗∗^
	60-month	55.9 ± 0.8 (5201)	58.4 ± 0.7 (6232)^∗∗∗^	62.4 ± 0.7 (6783)^∗∗∗^	68.0 ± 0.6 (9052)^∗∗∗^

Caucasian	6-month	91.6 ± 0.2 (15960)	92.4 ± 0.2 (17189)^∗∗∗^	92.4 ± 0.2 (17293)	93.6 ± 0.2 (26318)^∗∗∗^
	12-month	81.9 ± 0.3 (15960)	83.6 ± 0.3 (17189)^∗∗∗^	84.9 ± 0.3 (17293)^∗∗∗^	87.5 ± 0.2 (26318)^∗∗∗^
	24-month	69.8 ± 0.4 (15960)	72.3 ± 0.4 (17189)^∗∗∗^	75.0 ± 0.4 (17293)^∗∗∗^	79.2 ± 0.3 (26318)^∗∗∗^
	36-month	63.7 ± 0.4 (15960)	65.9 ± 0.4 (17189)^∗∗∗^	69.8 ± 0.4 (17293)^∗∗∗^	74.4 ± 0.3 (26318)^∗∗∗^
	48-month	59.5 ± 0.4 (15960)	61.5 ± 0.4 (17189)^∗∗∗^	66.1 ± 0.4 (17293^∗∗∗^	71.2 ± 0.3 (26318)^∗∗∗^
	60-month	56.2 ± 0.5 (15960)	58.5 ± 0.4 (17189)^∗∗∗^	63.3 ± 0.4 (17293)^∗∗∗^	68.8 ± 0.4 (26318)^∗∗∗^

African American	6-month	87.0 ± 0.7 (2757)	88.5 ± 0.6 (3276)^∗∗∗^	86.9 ± 0.6 (3264)^∗∗∗^	87.8 ± 0.6 (3448)^∗∗∗^
	12-month	72.9 ± 0.9 (2757)	74.1 ± 0.8 (3276)^∗∗∗^	75.1 ± 0.8 (3264)^∗∗∗^	78.0 ± 0.7 (3448)^∗∗∗^
	24-month	57.2 ± 1.0 (2757)	57.5 ± 0.9 (3276)^∗∗∗^	60.4 ± 0.9 (3264)^∗∗∗^	64.8 ± 0.9 (3448)^∗∗∗^
	36-month	48.1 ± 1.0 (2757)	49.3 ± 0.9 (3276)^∗∗∗^	53.8 ± 0.9 (3264)^∗∗∗^	57.7 ± 0.9 (3448)^∗∗∗^
	48-month	44.0 ± 1.0 (2757)	44.6 ± 1.0 (3276)^∗∗∗^	49.7 ± 1.0 (3264)^∗∗∗^	53.5 ± 1.0 (3448)^∗∗∗^
	60-month	40.7 ± 1.0 (2757)	41.5 ± 1.0 (3276)^∗∗∗^	46.1 ± 1.0 (3264)^∗∗∗^	49.9 ± 1.0 (3448)^∗∗∗^

Other	6-month	93.2 ± 1.2 (522)	93.1 ± 0.7 (1459)	94.2 ± 0.5 (2194)^∗∗∗^	94.1 ± 0.5 (2942)^∗∗∗^
	12-month	84.6 ± 1.6 (522)	84.5 ± 1.0 (1459)	87.1 ± 0.8 (2194)^∗∗∗^	87.5 ± 0.6 (2942)^∗∗∗^
	24-month	73.7 ± 2.0 (522)	74.8 ± 1.2 (1459)^∗∗∗^	77.0 ± 1.0 (2194)^∗∗∗^	77.9 ± 0.8 (2942)^∗∗∗^
	36-month	68.0 ± 2.2 (522)	67.5 ± 1.3 (1459)^∗∗∗^	70.4 ± 1.0 (2194)^∗∗∗^	73.1 ± 0.9 (2942)^∗∗∗^
	48-month	62.0 ± 2.3 (522)	62.0 ± 1.4 (1459)	66.2 ± 1.1 (2194)^∗∗∗^	69.3 ± 0.9 (2942)^∗∗∗^
	60-month	58.2 ± 2.3 (522)	58.8 ± 1.4 (1459)^∗∗∗^	63.0 ± 1.1 (2194)^∗∗∗^	66.8 ± 1.0 (2942)^∗∗∗^

Low-poverty	6-month	92.1 ± 0.3 (9211)	92.3 ± 0.3 (10373)^∗∗∗^	92.6 ± 0.3 (12034)^∗∗∗^	93.8 ± 0.2 (19563)^∗∗∗^
	12-month	82.3 ± 0.4 (9211)	83.8 ± 0.4 (10373)^∗∗∗^	84.9 ± 0.4 (12034)^∗∗∗^	88.0 ± 0.3 (19563)^∗∗∗^
	24-month	69.5 ± 0.5 (9211)	72.2 ± 0.5 (10373)^∗∗∗^	75.1 ± 0.4 (12034)^∗∗∗^	79.7 ± 0.3 (19563)^∗∗∗^
	36-month	63.6 ± 0.6 (9211)	65.6 ± 0.5 (10373)^∗∗∗^	69.9 ± 0.5 (12034)^∗∗∗^	75.0 ± 0.4 (19563)^∗∗∗^
	48-month	59.4 ± 0.6 (9211)	61.1 ± 0.5 (10373)^∗∗∗^	66.2 ± 0.5 (12034)^∗∗∗^	71.8 ± 0.4 (19563)^∗∗∗^
	60-month	56.4 ± 0.6 (9211)	58.2 ± 0.6 (10373)^∗∗∗^	63.2 ± 0.5 (12034)^∗∗∗^	69.5 ± 0.4 (19563)^∗∗∗^

Medium-poverty	6-month	89.9 ± 0.3 (9576)	91.3 ± 0.3 (10984)^∗∗∗^	90.9 ± 0.3 (10098)^∗∗∗^	92.1 ± 0.3 (12568)^∗∗∗^
	12-month	79.1 ± 0.4 (9576)	80.8 ± 0.4 (10984)^∗∗∗^	82.3 ± 0.4 (10098)^∗∗∗^	84.7 ± 0.3 (12568)^∗∗∗^
	24-month	66.3 ± 0.5 (9576)	68.4 ± 0.5 (10984)^∗∗∗^	70.9 ± 0.5 (10098)^∗∗∗^	75.2 ± 0.4 (12568)^∗∗∗^
	36-month	59.3 ± 0.6 (9576)	61.5 ± 0.5 (10984)^∗∗∗^	64.9 ± 0.5 (10098)^∗∗∗^	69.6 ± 0.5 (12568)^∗∗∗^
	48-month	55.0 ± 0.6 (9576)	57.0 ± 0.5 (10984)^∗∗∗^	61.0 ± 0.5 (10098)^∗∗∗^	66.0 ± 0.5 (12568)^∗∗∗^
	60-month	51.5 ± 0.6 (9576)	53.8 ± 0.5 (10984)^∗∗∗^	58.1 ± 0.6 (10098)^∗∗∗^	63.3 ± 0.5 (12568)^∗∗∗^

High-poverty	6-month	92.6 ± 1.3 (511)	93.6 ± 1.1 (581)^∗∗∗^	91.7 ± 1.1 (692)^∗∗∗^	89.8 ± 1.1 (825)^∗∗∗^
	12-month	84.1 ± 1.8 (511)	82.4 ± 1.7 (581)^∗∗∗^	85.2 ± 1.5 (692)^∗∗∗^	80.3 ± 1.5 (825)^∗∗∗^
	24-month	75.6 ± 2.1 (511)	71.3 ± 2.1 (581)^∗∗∗^	74.7 ± 1.8 (692)^∗∗∗^	70.6 ± 1.7 (825)^∗∗∗^
	36-month	69.2 ± 2.3 (511)	65.6 ± 2.2 (581)^∗∗∗^	68.4 ± 2.0 (692)^∗∗∗^	65.5 ± 1.8 (825)^∗∗∗^
	48-month	67.3 ± 2.5 (511)	60.6 ± 2.3 (581)^∗∗∗^	64.8 ± 2.1 (692)^∗∗∗^	61.2 ± 2.0 (825)^∗∗∗^
	60-month	63.8 ± 2.6 (511)	58.0 ± 2.4 (581)^∗∗∗^	61.5 ± 2.2 (692)^∗∗∗^	58.0 ± 2.1 (825)^∗∗∗^

Localized	6-month	97.3 ± 0.2 (7945)	98.7 ± 0.2 (8767)^∗∗∗^	98.2 ± 0.2 (8334)^∗∗∗^	98.4 ± 0.2 (7612)^∗∗∗^
	12-month	93.5 ± 0.3 (7945)	95.6 ± 0.3 (8767)^∗∗∗^	95.9 ± 0.3 (8334)^∗∗∗^	96.8 ± 0.3 (7612)^∗∗∗^
	24-month	87.2 ± 0.5 (7945)	89.8 ± 0.4 (8767)^∗∗∗^	91.0 ± 0.4 (8334)^∗∗∗^	93.5 ± 0.4 (7612)^∗∗∗^
	36-month	82.8 ± 0.5 (7945)	85.4 ± 0.5 (8767)^∗∗∗^	87.7 ± 0.5 (8334)^∗∗∗^	91.1 ± 0.4 (7612)^∗∗∗^
	48-month	79.2 ± 0.6 (7945)	81.6 ± 0.5 (8767)^∗∗∗^	84.9 ± 0.5 (8334)^∗∗∗^	88.8 ± 0.5 (7612)∗∗∗
	60-month	75.9 ± 0.6 (7945)	79.2 ± 0.6 (8767)^∗∗∗^	82.4 ± 0.6 (8334)^∗∗∗^	87.4 ± 0.6 (7612)^∗∗∗^

Regional	6-month	89.5 ± 0.4 (7524)	89.7 ± 0.3 (9661)^∗∗∗^	90.6 ± 0.3 (10712)^∗∗∗^	93.3 ± 0.2 (12137)^∗∗∗^
	12-month	76.2 ± 0.5 (7524)	76.6 ± 0.5 (9661)^∗∗∗^	80.0 ± 0.4 (10712)^∗∗∗^	85.9 ± 0.3 (12137)^∗∗∗^
	24-month	59.2 ± 0.6 (7524)	60.4 ± 0.5 (9661)^∗∗∗^	66.2 ± 0.5 (10712)^∗∗∗^	75.9 ± 0.4 (12137)^∗∗∗^
	36-month	50.9 ± 0.6 (7524)	52.1 ± 0.5 (9661)^∗∗∗^	59.2 ± 0.5 (10712)^∗∗∗^	70.5 ± 0.5 (12137)^∗∗∗^
	48-month	45.9 ± 0.6 (7524)	46.7 ± 0.6 (9661) ^∗∗∗^	54.5 ± 0.5 (10712)^∗∗∗^	67.4 ± 0.5 (12137)^∗∗∗^
	60-month	42.3 ± 0.6 (7524)	43.2 ± 0.6 (9661)^∗∗∗^	51.2 ± 0.5 (10712)^∗∗∗^	65.1 ± 0.5 (12137)^∗∗∗^

Distant	6-month	74.6 ± 0.9 (2213)	75.3 ± 1.0 (2114)^∗∗∗^	76.2 ± 0.9 (2211)^∗∗∗^	83.2 ± 0.6 (3906)^∗∗∗^
	12-month	53.5 ± 1.1 (2213)	54.9 ± 1.1 (2114)^∗∗∗^	59.1 ± 1.1 (2211)^∗∗∗^	69.5 ± 0.8 (3906)^∗∗∗^
	24-month	34.8 ± 1.1 (2213)	36.2 ± 1.1 (2114)^∗∗∗^	42.3 ± 1.1 (2211)^∗∗∗^	55.3 ± 0.8 (3906)^∗∗∗^
	36-month	26.4 ± 1.0 (2213)	28.6 ± 1.0 (2114)^∗∗∗^	36.2 ± 1.1 (2211)^∗∗∗^	47.7 ± 0.9 (3906)^∗∗∗^
	48-month	23.1 ± 1.0 (2213)	25.1 ± 1.0 (2114)^∗∗∗^	32.8 ± 1.1 (2211)^∗∗∗^	43.3 ± 0.9 (3906)^∗∗∗^
	60-month	21.3 ± 0.9 (2213)	22.3 ± 1.0 (2114)^∗∗∗^	29.7 ± 1.0 (2211)^∗∗∗^	40.3 ± 0.9 (3906)^∗∗∗^

RSR: relatively survival rate; SEM: standard error of the mean. ^∗∗∗^*P* < 0.0001 for comparisons with the previous decade.

## Data Availability

All the data in the current study are publicly available in the Surveillance, Epidemiology, and End Results database (https://seer.cancer.gov/).

## Supplementary Materials

Supplementary Materials Supplementary figure 1: trends in overall survival by Kaplan-Meier survival analyses for head and neck cancer patients in SEER nine registries over past four decades in all cases (A), by races (C, E, G), and by age groups (B, D, F, H). Supplementary figure 2: trends in overall survival by Kaplan-Meier survival analyses for head and neck cancer patients in SEER nine registries over past four decades by sexes (A, B), by socioeconomic status (C, E, G), and by stages (D, F, H).
