# Corrigendum to “Epidemiological Trends of Head and Neck Cancer: A Population-Based Study”

**DOI:** 10.1155/2021/9758328

**Published:** 2021-11-24

**Authors:** Kangwen Guo, Weiliang Xiao, Xinggui Chen, Zhenying Zhao, Yuanxiong Lin, Ge Chen

**Affiliations:** ^1^Department of Radiotherapy, Central Hospital of Guangdong Nongken, Zhanjiang, China; ^2^Department of Intervention, The First Affiliated Hospital of Guangzhou University of Chinese Medicine, Guangzhou, China; ^3^Cancer Center, Affiliated Hospital of Guangdong Medical University, Zhanjiang, China; ^4^Department of Pharmacy, Tianjin Union Medical Center, Tianjin, China

In the article titled “Epidemiological Trends of Head and Neck Cancer: A Population-Based Study” [1], the affiliation order is incorrect.

The corrected affiliation is shown in the author information above.
