# Supplementary material for: Epidemiological Trends of Head and Neck Cancer: A Population-Based Study
Source: Biomed Res Int. 2021 Jul 14;2021:1738932. doi: 10.1155/2021/1738932 (PMC8294963; doi:10.1155/2021/1738932)
Supplement: Supplementary Materials — Supplementary figure 1: trends in overall survival by Kaplan-Meier survival analyses for head and neck cancer patients in SEER nine registries over past four decades in all cases (A), by races (C, E, G), and by age groups (B, D, F, H). Supplementary figure 2: trends in overall survival by Kaplan-Meier survival analyses for head and neck cancer patients in SEER nine registries over past four decades by sexes (A, B), by socioeconomic status (C, E, G), and by stages (D, F, H). [file 1738932.f1.docx]

**Epidemiological Trends of Head and Neck Cancer, a Population-based Study**

Kangwen Guo^1*†,^ Weiliang Xiao^2*^, Xinggui Chen^3*^, Zhenying Zhao^4^, Yuanxiong Lin^1^, Ge Chen^1^

^1^Department of Radiotherapy, Central hospital of Guangdong Nongken, Zhanjiang, China;

^2^Department of Intervention, The First Affiliated Hospital of Guangzhou University of Chinese Medicine, Guangzhou, China;

^3^Cancer Center, Affiliated Hospital of Guangdong Medical University, Zhanjiang, China;

^4^Department of Pharmacy, Tianjin Union Medical Center, Tianjin, China;

^*^These authors contributed equally to the study.

^†^Correspondence to: Kangwen Guo, M.D., Department of Radiotherapy, Central hospital of Guangdong Nongken, Zhanjiang, China, email: [guokangwen90@163.com](mailto:guokangwen90@163.com);

**Supplementary figure 1.**


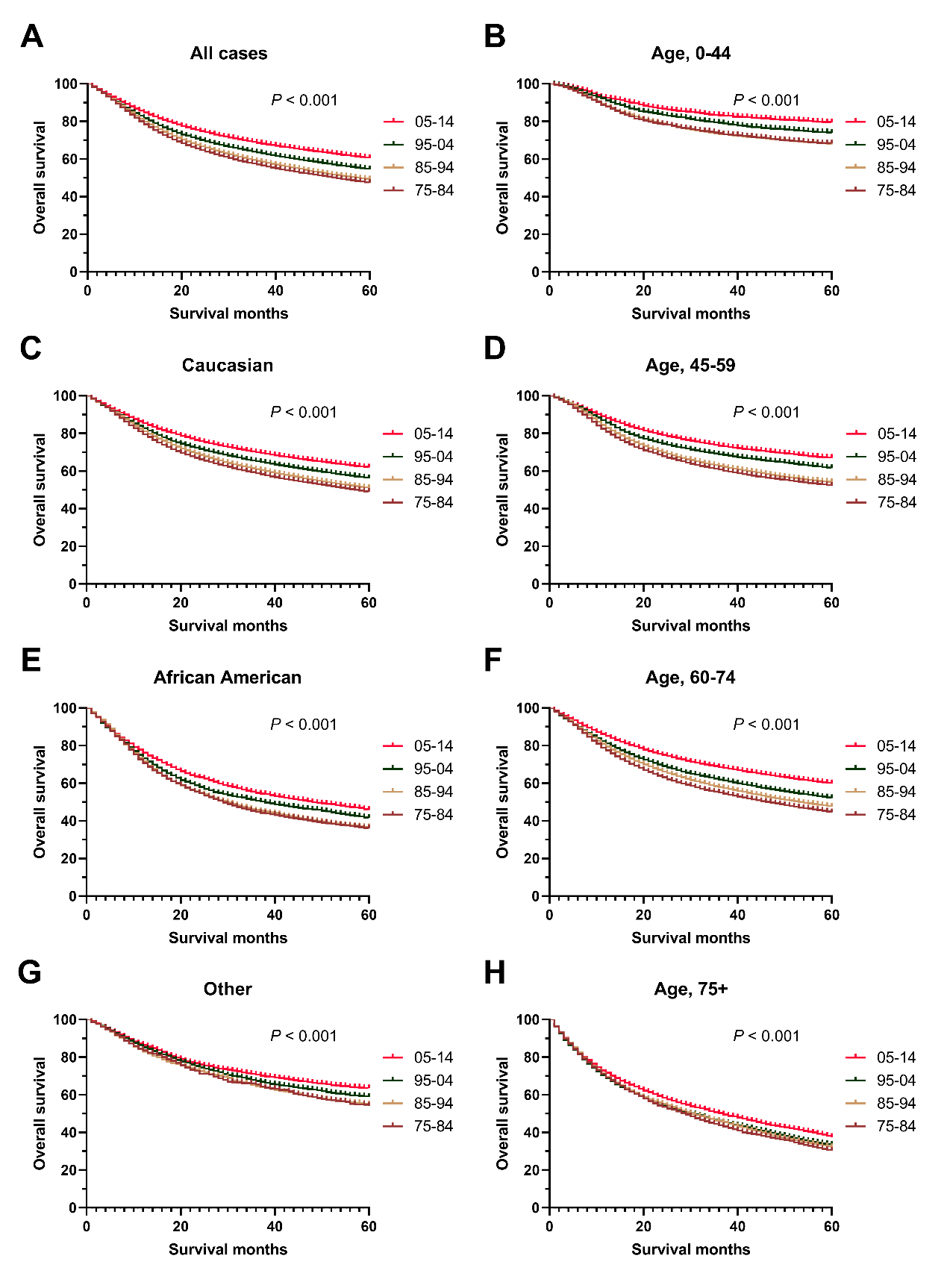


**Supplementary figure 2.**


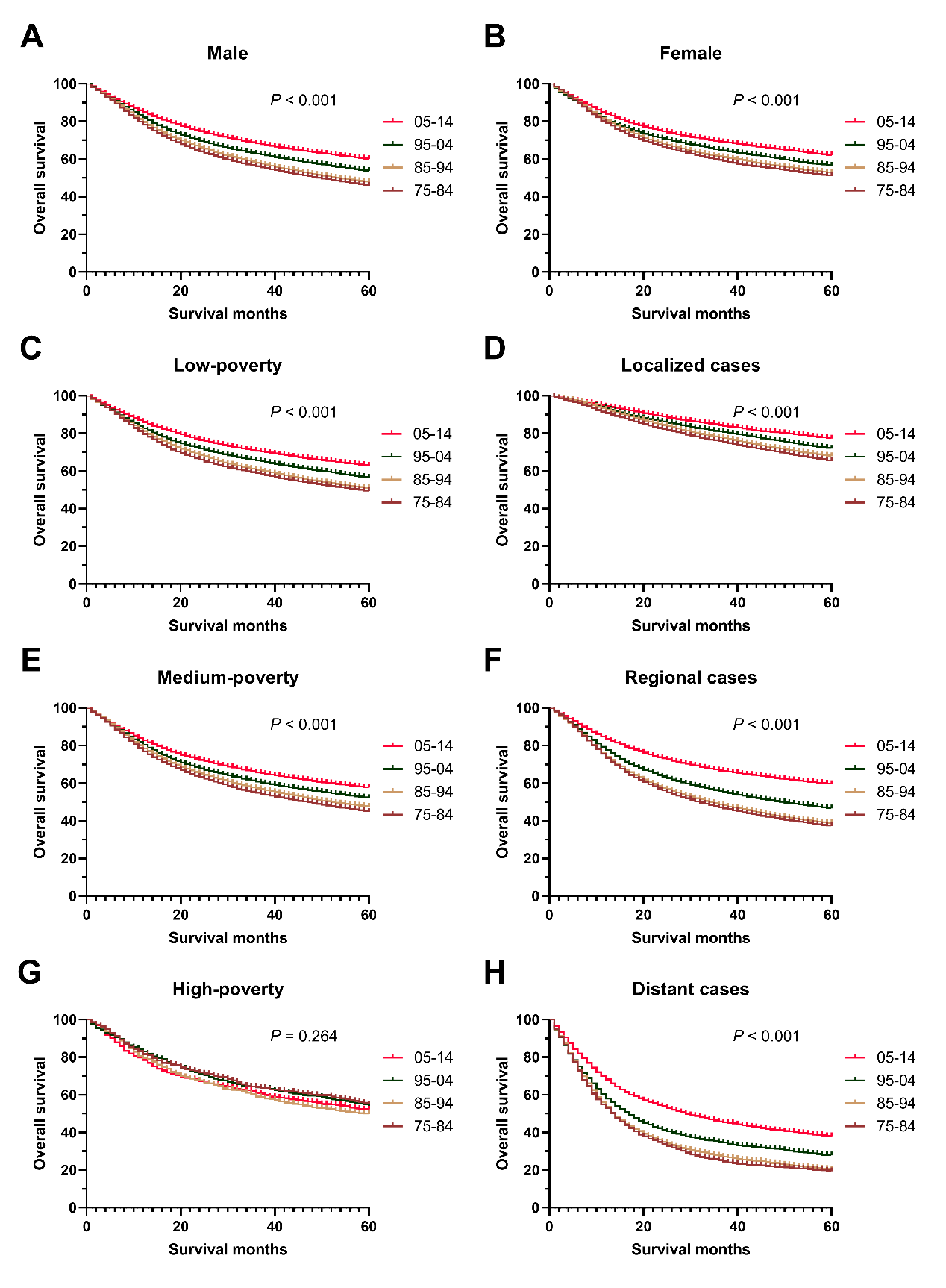


Description of supplementary file: This supplementary file contains two supplementary figures.

**Supplementary figure 1.** Trends in overall survival by Kaplan-Meier survival analyses for head and neck cancer patients in SEER nine registries over past four decades in all cases (A), by races (C, E, G) and by age groups (B, D, F, H).

**Supplementary figure 2.** Trends in overall survival by Kaplan-Meier survival analyses for head and neck cancer patients in SEER nine registries over past four decades by sexes (A, B), by socioeconomic status (C, E, G) and by stages (D, F, H).
